# Human-inspired strategies for controlling swarm systems

**DOI:** 10.1098/rsta.2024.0147

**Published:** 2025-01-30

**Authors:** Patrick Nalepka, Gaurav Patil, Rachel W. Kallen, Michael J. Richardson

**Affiliations:** ^1^ Performance and Expertise Research Centre, Macquarie University, Sydney NSW 2109, Australia; ^2^ School of Psychological Sciences, Macquarie University, Sydney NSW 2109, Australia

**Keywords:** human performance, multi-agent coordination, human–machine interaction

## Abstract

The control of swarms has emerged as a paradigmatic example of human-autonomy teaming. This review focuses on understanding human coordination behaviours, while controlling evasive autonomous agents, to inform the design of human-compatible teammates. We summarize the solutions employed by human dyads, as well as the verbal communication and division of labour strategies observed in four-person teams using virtual simulations. Additionally, we provide an overview of the design of artificial agents that replicate human-like dynamics using task-dynamical models, and which can be integrated into human-autonomy teams. Finally, we conclude with open questions regarding the preservation of situation awareness and trust within human-autonomous swarming teams.

This article is part of the theme issue ‘The road forward with swarm systems’.

## 1. Introduction

The concept of ‘swarms’ elicits imagery of amazing displays of collective behaviour, such as the transportation of food by ant colonies, the shoaling of fish, the flocking of birds [1] or bio-inspired multi-agent robotics [2]. What defines these systems as swarms is the idea that simple agents, employing simple rules, can interact to produce complex patterns of behaviour at the collective level.

The swarms concept has also been applied to understanding human collective behaviour, often within larger organizational structures. Examples include agent-based models that illustrate how severe racial segregation can emerge from agents displaying minor preferences in where to live [3], the effect of social network structure on collective memory [4] or the emergence of lanes in human crowd navigation [5]. However, these same principles can be applied to understanding the coordinative structures that emerge in smaller social units, such as the scale of dyadic or small group interactions.

## 2. Human small groups as swarms

In the perceptual control of action in humans, research within the task-dynamics (also referred to as behavioural dynamics) [6,7] framework has shown that simple decisions made by humans during social interactions can lead to emergent and complex behaviours [8]. This includes implicit forms of coordination such as crowd navigation [9] or the mirroring of actions [10], but also explicitly in collaborative tasks such as sorting and passing objects [11], and competitive sports tasks like football [12] or air hockey [13].

The task-dynamics framework is a task-oriented approach that assumes that goal-directed human behaviour is self-organized. Specifically, that the many degrees of freedom of the human perceptual-motor system become constrained to control information generated by interactions with environmental surfaces, objects and events [7]. Importantly, the coordinated behaviours or structures that result are low-dimensional, matching the (low) dimensionality of the control problem itself [14]. For example, to run to catch a fly ball requires moving so that the ball’s trajectory is optically linear along the transverse and sagittal planes [15]. This approach does not specify how each individual limb must act, but that the overall system is constrained by this superordinate structure, which affords an overall flexibility for human bodies to adapt and re-organize in case of perturbation or injury [16].

Task-dynamic models have been defined using nonlinear dynamical systems due to several convenient properties of such systems that emulate features of human perceptual-motor control. Specifically, elementary dynamical systems can be defined using systems that describe fixed-point and limit cycle attractors, which can be composed to generate more complex behaviours. Similarly, research in human motor control has demonstrated that complex human movements can be decomposed into a corresponding set of *motor primitives*, consisting of discrete (point attractor) and rhythmic (limit cycle) motions, which can be modelled as nonlinear dynamical systems, referred to as *dynamical motor primitives* (DMPs) [17]. Implementing DMPs is one common approach to controlling robotic systems, for example [18].

Similar to how simple agents following simple rules can give rise to complex collective phenomena due to interactions among agents (e.g. Reynold’s bird flocking model [19]), interactions among humans who each are implementing their own control policy can give rise to collective patterns of behaviour, and new strategies for collaboration [20]. The next section will highlight one example of this from our own research.

## 3. The human shepherding paradigm

**Figure 1.**
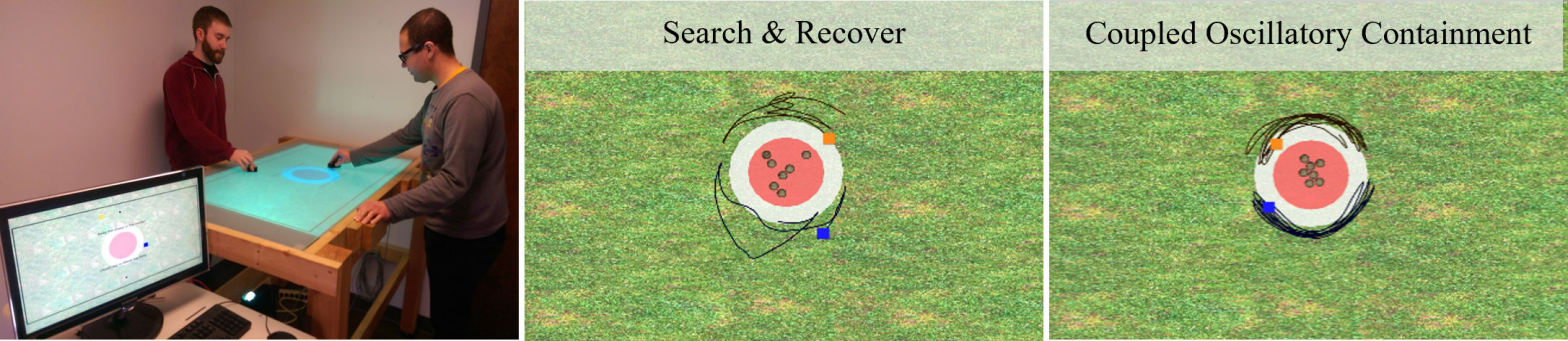
Human shepherding game environment and resultant strategies from [20]. Participants held motion sensors that moved their respective herding agents (the blue and orange square in the middle, right panels). Participants were instructed to keep all target agents (the brown spheres) within the red circular containment location. The middle and right panels demonstrate the two strategies human dyads used when completing the task. See text for more details. Figure adapted from [25].

The human ‘shepherding game’ is a research paradigm used to explore social perceptual-motor coordination and problem-solving among dyads [20], and more recently small groups [21,22]. Additionally, solutions to the ‘shepherding problem’ have been well investigated in the engineering community as an approach to control swarm systems (e.g. [23], for a review). Inspired by the steering behaviours of sheepdogs to control sheep flocks [24], the game involves participants working together to control a set of autonomous agents (i.e. target agents or TAs), and their movements towards a centralized containment location (see figure 1). Participants accomplished this goal by controlling a herding agent (HA) that can repel the TAs when in close proximity. In its original formulation, players controlled their HA via hand movements across a tabletop display [20]. In addition to being responsive to players’ movements, the TAs exhibit Brownian motion when left unperturbed, requiring participants to continuously interact with the TAs to ensure they remain within the containment location. Task success is defined by participants keeping all TAs within the containment location for a prescribed period (e.g. 70% of a 1-min trial). The game was won if participants could meet the success criteria on multiple attempts (e.g. success on eight attempts).

Unlike the behaviours observed in natural sheep flocks, where sheep exhibit herd flocking behaviour resulting in clumping [26], the TAs in this game did not have this coupling. Instead, the TAs could only interact with each other through collisions when fleeing the participants’ HAs. Despite this difference with natural systems and the increased task difficulty it entails, the strategies humans learned to employ were remarkably similarity to the strategies employed by sheepdogs [24] and other predatory animals during collaborative hunting [27].

### (a) Dyadic collaborative problem-solving

Using naive participants, human dyads completing the shepherding task learned to implement two strategies or behavioural modes [20]. The first, referred to as search & recover (S&R), is an intuitive strategy that is defined by participants first creating equal partitions of the game environment, and then each participant pursuing the TA that is farthest from the containment location. The decision determining the farthest agent could be made using Euclidean distance information alone [28], or could also factor in TA velocity information to anticipate where TAs will be in the future [25].

The S&R strategy is sufficient to complete the task within certain, easier task difficulty settings such as when there are a small number of TAs present [20], or their evasive capabilities are limited [25]. When task difficulty increased, however, participants who relied on the S&R strategy were more likely to fail the task.

Despite these more difficult task configurations, a subset of participants learn to employ a novel containment strategy referred to as coupled oscillatory containment (COC) [20]. The defining feature of this strategy is participants learning to produce rhythmic, oscillatory movements between multiple TAs to collectively shepherd them towards the containment location and, once contained, to continue to oscillate around the contained herd with their partner, resulting in the formation of a spatial-temporally defined barrier [20] (see figure 1). Participants who discover and transition to the COC strategy achieve near optimal levels of task performance.

The ‘coupled’ component of the strategy’s name is due to the observation that when both participants engage in these oscillatory behaviours, their movements become informationally coupled to each other, resulting in the emergence of the two commonly observed patterns of rhythmic coordination observed in biological systems [29]. Namely, the observation of in-phase and anti-phase relative phase behaviours [10,29]. Note that the particular pattern of relative phase participants adopt functionally makes no difference in the context of this task. All that is required is that both participants produce oscillatory movements above a certain frequency, which is sufficient to keep the TAs contained. The observed emergent patterns of in-phase and anti-phase rhythmic coordination are simply an unintentional, self-organized consequence of human-coupled oscillatory systems.

The differences between COC-discovering and non-discovering dyads may either be due to differences in the decision policies participants employ when attempting the task [30], differences in participants’ ability to detect the strategy as a latent property of the task [25], or differences in the coupling between participants [31]. Collectively, the research suggests the following: differences in individual decision policies, as well as incidental interactions between participants, result in the emergence of movements that resemble oscillatory-like features. When generated, dyads who will discover the COC strategy may be able to detect these features, resulting in the insight that these oscillatory movements can be utilized explicitly to complete the task.

### (b) Task-dynamic model of human shepherding

**Figure 2.**
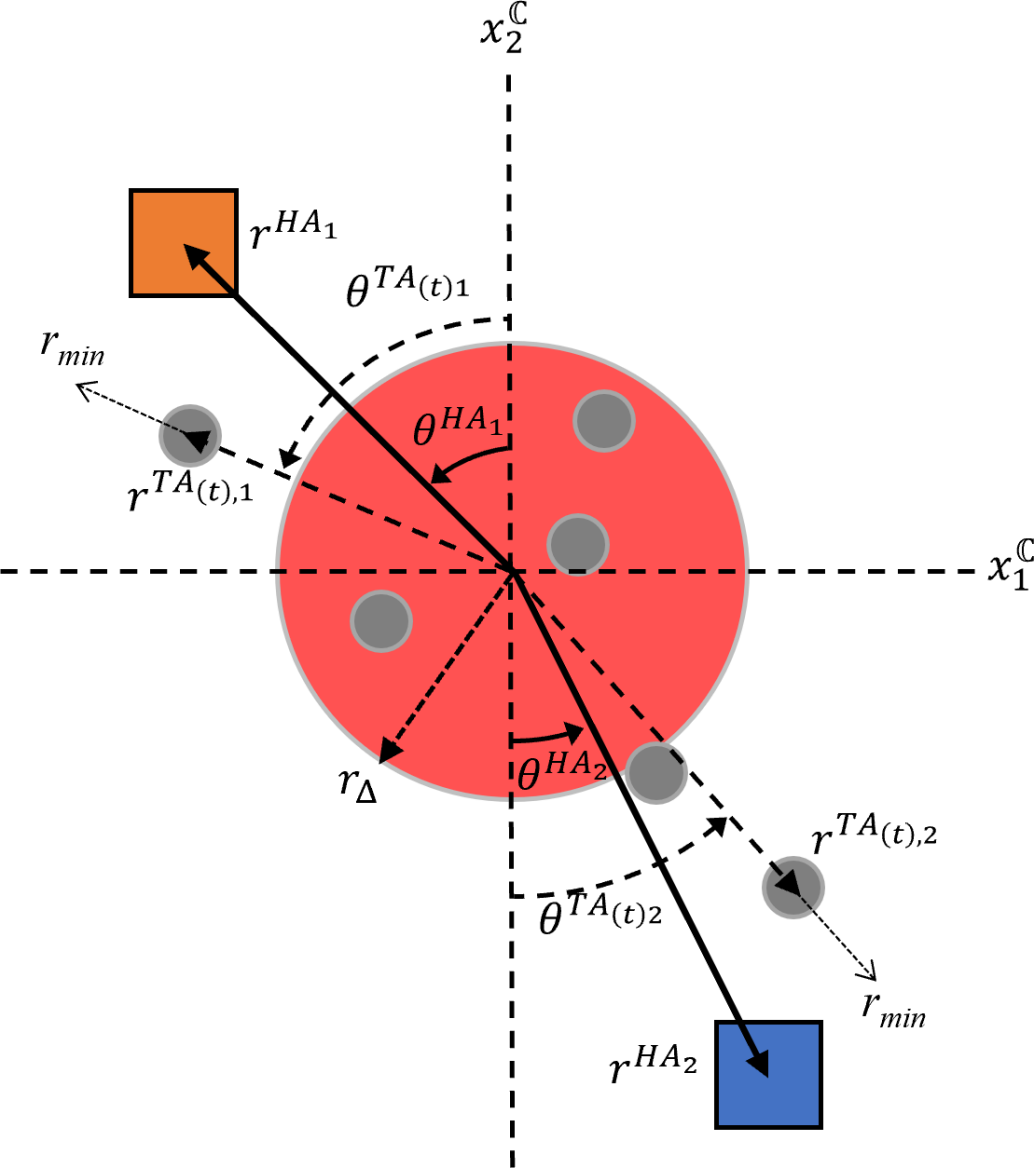
Pictorial representation of the shepherding task-dynamic model. The position of herding agent (HA) i (here, i=1,2) is defined in a polar coordinate task-space ($r^{HA_{i}},\theta^{HA_{i}}$), with the origin being the centre of the red containment location. At each time t, each HA moves towards the position of the target agent (TA) ($r^{TA_{(t),i}},\theta^{TA_{(t),i}}$) using the TA selection rule defined in equation (3.1). See text for more details. Figure adapted from [25].

The behavioural strategies human participants discovered during the shepherding task can be captured using a heuristic model that (i) defines the decisions participants make using an algorithmic rule; and (ii) once a particular decision is made, generates the motions necessary to carry out that decision using a system of nonlinear dynamical equations that control the radial and angular components of the participants’ movements (see figure 2), where the polar origin is the centre of the containment location, serving as the goal for where the TAs must be shepherded.

First, the algorithmic rule that determines which TA a player pursues at time t is the following:


(3.1) $$\{TA_{(t),i}\in H_{i}|\max(\|x^{TA_{(t),i}}+{\dot{x}}^{TA_{(t),i}}\Delta t\|)\},$$

where at time t, participant i considers the subset of TAs that are closer to themselves than their partner ($H_{i}$). From this subset, participant i chooses the TA that is farthest from the containment location currently (if Δt=0 [28]) or in the future (Δt≠0 [25]), where 𝒙 represents the TA’s Cartesian position and velocity ($\dot{𝒙}$) with respect to the containment location.

Second, the equations that capture the motions of participants are defined in equations (3.2) and (3.3):


(3.2) $$\begin{array}{ccc} & {\ddot{r}}^{HA_{i}}+b_{r}{\dot{r}}^{HA_{i}}+\epsilon_{r}\left(r^{HA_{i}}-\left(r^{TA_{(t),i}}+r_{min}\right)\right)=0 & \end{array}$$

(3.3) $${\ddot{\theta }}^{HA_{i}}+b_{\theta }{\dot{\theta }}^{HA_{i}}+\epsilon_{\theta }(\theta^{HA_{i}}-\theta^{TA_{(t),i}})=0,$$

where (3.2) and (3.3) are simple damped mass-spring oscillators with damping b and stiffness ϵ that minimizes the radial r and angular θ discrepancies between participant i HA’s current position and its goal, defined as $\left(r^{TA_{(t),i}}+r_{min},\theta^{TA_{(t),i}}\right)$, where $TA_{(t),i}$ is the selected TA from 2.1, and $r_{min}$ represents a radial offset to ensure the HA is positioned farther from the TA to ensure that the TA is repelled towards the containment location.

Equations (3.1), (3.2) and (3.3) are sufficient to generate S&R behaviour. However, (3.3) must be extended to capture the behavioural mode switching to self-sustaining oscillatory behaviour as observed in the COC strategy. This can be done by extending (3.3) to include additional nonlinear terms [32]. Further, to replicate the emergent in-phase and anti-phase patterns of rhythmic coordination between participant i and their partner j, an additional coupling term can be included [33]:


(3.4) $$\begin{array}{r} {\ddot{\theta }}^{HA_{i}}+b_{\theta }{\dot{\theta }}^{HA_{i}}+\beta_{\theta }({\dot{\theta }}^{HA_{i}}{)}^{3}+\gamma_{\theta }(\theta^{HA_{i}}{)}^{2}{\dot{\theta }}^{HA_{i}}+\epsilon_{\theta }(\theta^{HA_{i}}-\theta^{TA_{(t)i}})\\ =({\dot{\theta }}^{HA_{i}}-{\dot{\theta }}^{HA_{j}})(A+B(\theta^{HA_{i}}-\theta^{HA_{j}}{)}^{2}). \end{array}$$

The inclusion of the Rayleigh ($\beta_{\theta }({\dot{\theta }}^{HA_{i}}{)}^{3}$) and van der Pol ($\gamma_{\theta }(\theta^{HA_{i}}{)}^{2}$) escapement terms allow for the emergence of oscillatory behaviour [28,32]. The coupling term $\left({\dot{\theta }}^{HA_{i}}-{\dot{\theta }}^{HA_{j}})(A+B(\theta^{HA_{i}}-\theta^{HA_{j}}{)}^{2}\right)$) enables stable in-phase and anti-phase to occur, where parameters A and B index coupling strength, with both behavioural modes possible when |4B|>|A| [33].

Equation (3.4) is capable of displaying both S&R and COC behaviour. When $b_{\theta }>0$, the dynamics of (3.4) exhibited similar point attractor dynamics as (3.3). However, when $b_{\theta }<0$, a Hopf bifurcation occurs [17], resulting in the destabilization of point attractor dynamics and the emergence of limit cycle, oscillatory dynamics at a constant frequency $\sqrt{\epsilon_{\theta }}$ (labelled ω in [32]).

When using (3.1), an additional decision heuristic is needed to indicate when transitions between S&R and COC should occur. This is done by defining the following dynamics equation for $b_{\theta }$:


(3.5) $$\dot{b_{\theta }}+\delta (b_{\theta }-\alpha (r^{TA_{(t),i}}-r_{\Delta }))=0,$$

where δ and α are constants, and $r_{\Delta }$ represents the critical crossing distance from the containment location for TAs to be considered sufficiently corralled, and thus the COC strategy is used to contain the TA herd.

The model described in Equations (3.1), (3.2), (3.4) and (3.5) has been validated in [28], which will be discussed in more detail in §4.

### (c) Solution similitude in other contexts

Before turning to review how task-dynamic models can be used to design human-compatible artificial agents, we next want to highlight how the above task-dynamic model, when emulating novice-level behaviour, can be used to understand the contextual emergence of collaborative problem-solving strategies in disparate contexts.

As mentioned previously, the strategies human dyads adopt when completing the task using hand movements along a tabletop display resemble the strategies adopted by other non-human systems in shepherding [24] or collaborative hunting [27,34] contexts. These animals and human participants vary drastically in their capabilities as well as the constraints imposed by the environments in which they operate. What is shared among these examples, however, is the task goal of corralling autonomous agents to a centralized location (to either earn research credits in the case of humans [20] or to catch prey). A solution shared across these systems is the utilization of encirclement behaviours that limits the escape options of the target agents.

**Figure 3.**
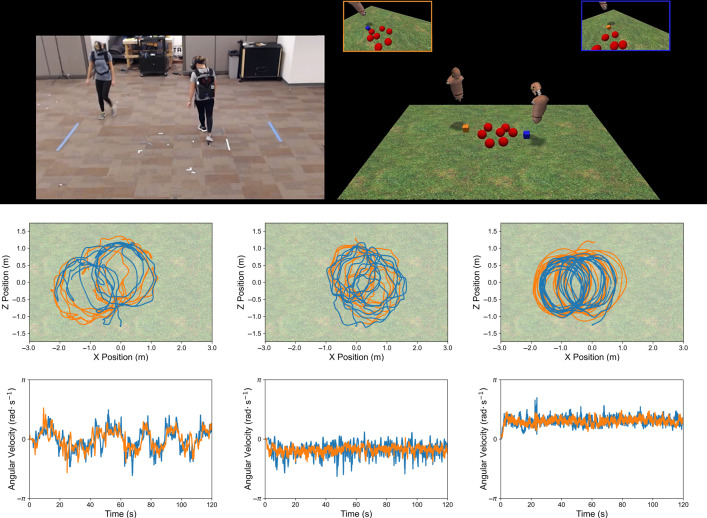
Task environment and resultant behaviours when humans solve a locomotion-based shepherding task in immersive virtual reality. Participants wore head-mounted virtual reality headsets connected to a computer worn as a backpack. Participants embodied floating avatars and could locomote about a large (6 × 3.48 m) environment to contain seven evasive target agents (TAs), which varied in their maximum evasive velocities. The middle and bottom third of the figure represent the observed behaviours of participants, as a function of TA maximal velocity (low, medium, high). As task difficulty increased, dyads were more likely to solve the task by circling in a fixed direction. See text for more details. Figure adapted from [25].

Inspired by these examples in natural systems, Nalepka *et al*. [25] presented the same shepherding problem to human participants. However, instead of using handheld controllers to control their HAs, participants interacted with the game elements by locomoting in a large virtual environment (see figure 3). Like the participants from [20], participants who were most successful discovered and exploited a strategy that involved encirclement behaviours. However, unlike previous work that implemented coordinated oscillatory behaviours, participants in this study [25] learned to solve the task by circling around the herd of autonomous agents in a fixed direction (see figure 3).

The different encirclement behaviours observed by human dyads between the tabletop video game and immersive virtual reality (VR) contexts can be understood as different expressions of the same generative model introduced in §3b. To illustrate, the model can first be modified to emulate novice, pre-discovery behaviours. This can be done by using equation (3.3) instead of (3.4). Agent-based simulations were conducted by [25] using this reduced model with the parameter constants adjusted to emulate the differential constraints acting upon participants in the different task settings. For example, the artificial agents were tuned to move quickly in the tabletop configuration, and more lethargic in the immersive VR environment configuration, to emulate the difference in effective mass, and therefore the time needed to translate across the game field.

**Figure 4.**
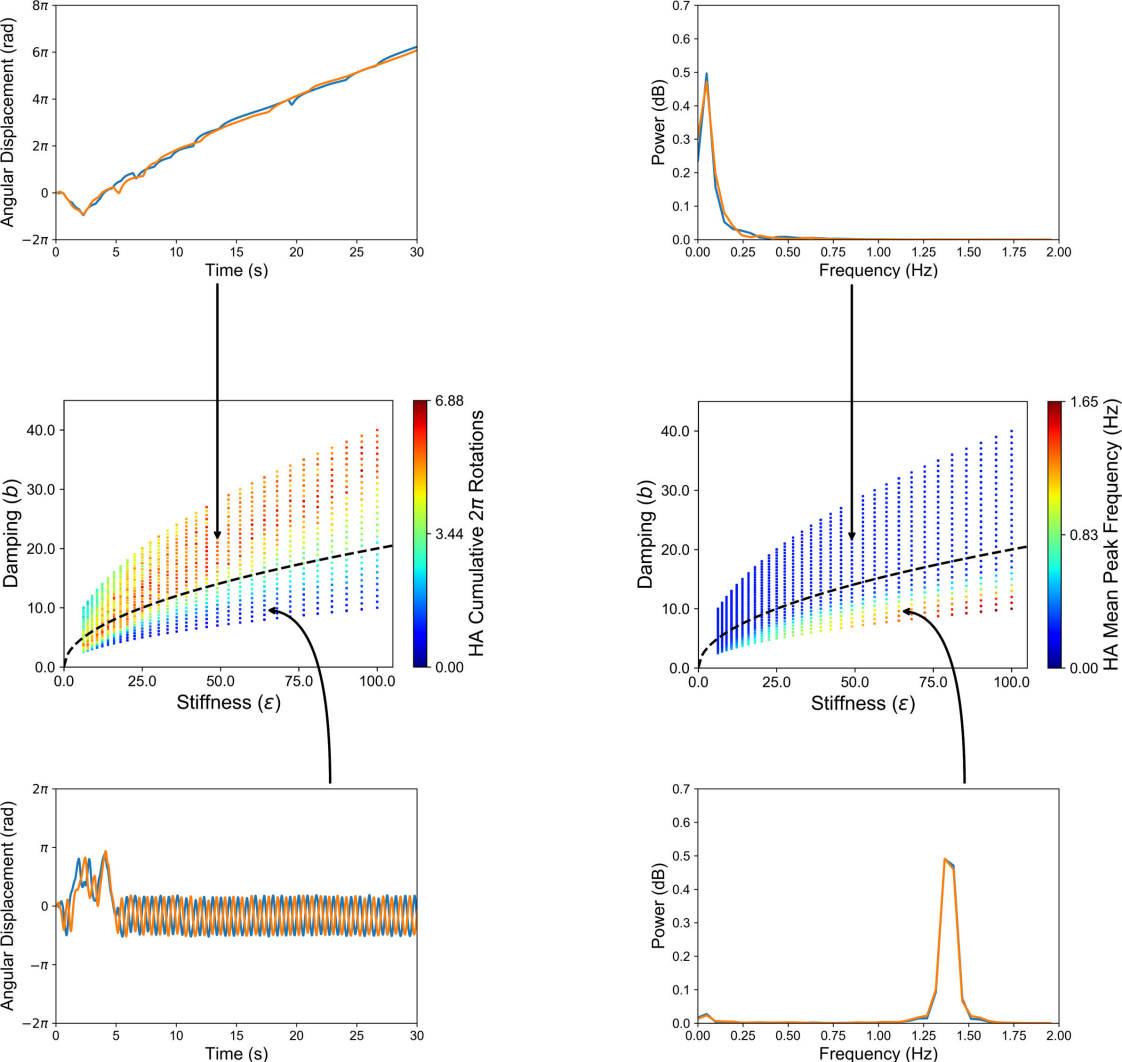
Agent-based model simulations results for shepherding task-dynamic model and different parameter constraints. Two different behaviours emerged as a function of changes to stiffness and damping (middle row), as illustrated by the cumulative 2π rotations (middle left) and the mean peak frequency of angular movement (middle right) by the simulated herding agents (HAs). The black dashed line in the middle panel represents critical damping, which satisfies the condition $\beta =2\sqrt{m\epsilon }$ (here, m=1kg). See text for more details. Figure adapted from [25].

When deployed, agents embodying the task-dynamic model introduced in §3b engaged in interactions with the environment that gave rise to emergent, oscillatory-like (when tuned to the tabletop environment) and circling-like (when tuned to the locomotive VR environment) behaviours (see figure 4). These behaviours were not ‘coded’ in these agents or the model, but were both an emergent consequence of the task’s dynamics, providing further evidence that effective strategies during social coordination can naturally emerge from the interaction of agents implementing simple decision policies. We now turn to how these agents can be employed to facilitate effective human–machine interaction.

## 4. Human–human compatible AI interaction

The previous research employing the shepherding task [20,25,28] observed that the behaviours adopted by human dyads can be modelled as low-dimensional dynamical systems, as introduced in §3b. Task-dynamic models can not only define the movement dynamics of individual agents but can also couple them to the dynamics of the environment and other agents (e.g. equation (3.4)). Incorporating the periodic and aperiodic rhythmic movement characteristics of humans, these models have been embedded within the control architectures of artificial agents, posing as virtual participants, to coordinate their actions with humans in a human-like manner.

Relevant to this article is the work by [28] whereby naive participants completed the tabletop shepherding task within a VR environment. In the experiments presented in [28], participants either interacted with other naive human participants, a human confederate who had expertise with the task, or a virtual artificial agent implementing the model described in §3b. All participants and their partners were embodied as similar virtual avatars. The results of these experiments showed that, when interacting with the virtual artificial agent, participants were able to solve the task and perform at levels similar to if they were working with a human expert. Further, evidence that the virtual agent emulated human-like dynamics was provided by participants in a post-session debriefing where 63% of participants believed their virtual partner to be another human participant, when in fact it was the artificial agent. Additionally, the participants interacting with the virtual artificial agent were able to display the same emergent patterns of rhythmic coordination observed in human–human dyads (in-phase and anti-phase oscillatory dynamics), and which have been observed during interpersonal rhythmic coordination more generally [10]. Follow-up work also demonstrated that participants can coordinate effectively with the agent not only in the original formulation of the task, but also in extended versions that required the transportation of objects between locations [35].

In addition to emulating human-like behaviours, these virtual artificial agents can also be used to improve collaborative problem-solving. Rigoli *et al*. [36] demonstrated that participants exposed to an agent embodying the task-dynamic model during training excelled at solving novel shepherding tasks with other human participants who received the same training. Participants who received this training performed at levels similar to or better than participants trained with a human expert. One important implication of these findings is that artificial agents can be integrated within social and team training exercises to provide trainees flexibility to undertake training on their schedule, while also reducing the costs associated with employing humans to pose as teammates for the training to be effectively conducted.

Thus far, our discussion of artificial agents consisted of control architectures involving dynamical models and heuristic rules. Machine-learning techniques, such as deep reinforcement learning (DRL), however, have made substantial progress in learning human-compatible models from experience. An illustrative example is the work of Carroll *et al*. [37], which demonstrated that reinforcement learning agents, when exposed to agents embodying learned models of human behaviour in a dyadic kitchen coordination task, developed policies that better aligned with human participants. Nalepka *et al*. [38] demonstrated that the dynamics of these agents similarly reflect types of interaction flexibility that is sought after in human–human teaming generally [39]. Importantly, agents that trained with agents that embody learned human models coordinated better with participants than if participants interacted with the learned human models directly or with DRL agents that were trained using self-play. This research demonstrates that reinforcement learning methods that do not consider the interaction styles that define human behaviour within simulation can fail to result in agents that can interact with humans effectively. Similarly, [36] showed that DRL agents developed action policies that, although superior in performance to humans during simulation, were significantly inferior to those of human experts and agents employing the shepherding task-dynamic model, when playing alongside humans.

A possible compromise between heuristic and machine-learned approaches is a hybrid, hierarchical approach [40]. Here, the decision policies of DRL agents can be constrained by using task-dynamic models as constraints for how these agents can navigate their environments. By reducing the complexity of the training problem to decisions only (e.g. which agent to pursue), the time required to train such agents predictably decreased, while also preserving human-like movement characteristics [41]. Despite this scaffolding, the decisions made by trained DRL agents during self-play did not align with those of their human partners. This was determined by simulating what the DRL agent would have selected if it was embodying the human participant [42]. However, similar to [37], if these hierarchical DRL-DMP agents were instead exposed to a partner embodying the heuristic task-dynamic model during training (as opposed to a similar DRL agent as done in traditional self-play), these agents exhibited better decision alignment with human novices.

The findings summarized in this section provide new opportunities to augment the DRL training paradigm with the inclusion of task-dynamic models that simulate the behaviours of humans. Such hybrid, hierarchical training can scaffold the behavioural policies of DRL agents to better coordinate with humans during human–machine interaction. In addition to fostering more human-aligned DRL agents, task-dynamic models can also be used to create synthetic datasets for supervised machine learning, to either supplement readily available human datasets, or to warm-start neural network training.

## 5. Human teaming during collaborative search

**Figure 5.**
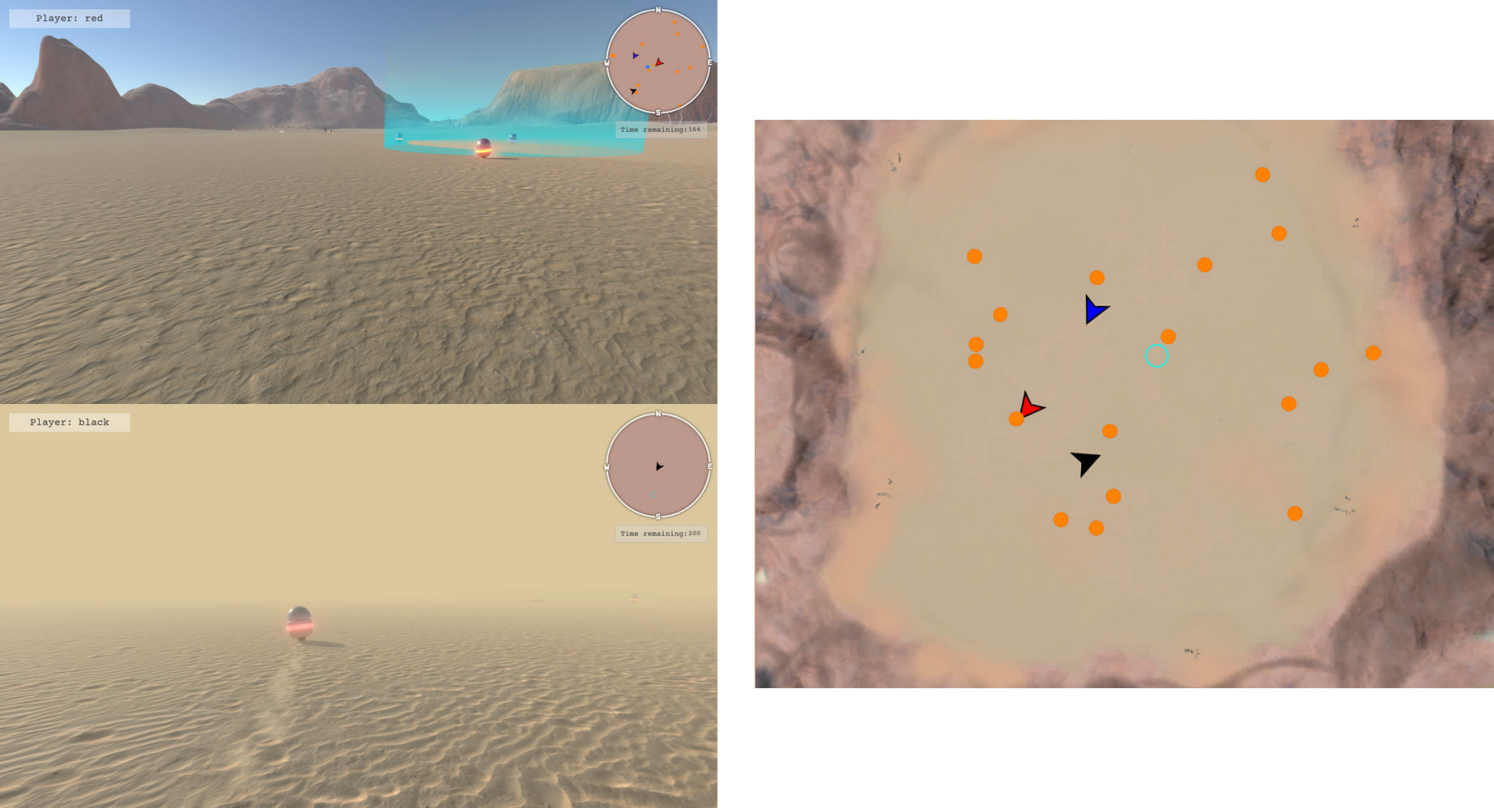
The shepherding task as a first-person multi-player video game. Participants used keyboard and mouse controls to navigate a large desert environment (approximately 600 m × 600 m) to locate and retrieve scattered autonomous agents and bring them to a cyan-coloured ring located in the centre of the environment. Participants worked as part of three- or four-person teams, and either played the role of *ground personnel* (left panel) or as an *operator* (right panel), who had a map view of the task. Participants were also exposed to environmental fog (left panel, bottom), making completion of the task difficult without access to a head-up display (the circular maps in the top right of the ground personnel’s view), or access to the operator who can give instructions. See text for more details. Figure adapted from [21].

Most recently, the shepherding paradigm has been extended to investigate team coordination in three- and four-person small groups [21,22]. Using keyboard and mouse controls, similar to first-person shooter video games, teams were required to coordinate their actions to search for and retrieve autonomous agents scattered throughout a large desert environment (see figure 5). The size of the game environment, and the potential for the presence of environmental fog to obscure vision, made it necessary for teams to rely on verbal communication, use head-up displays [21], or to rely on a remote operator who had sole access to veridical information about the environment [22], to divide labour and coordinate effectively to finish the task quickly.

Research to date using this extended game environment has documented the division of labour strategies that participants use to solve the task, as well as quantified the situation awareness [43] of teammates. Specifically, successful teams distributed workload by equally dividing the search space between team members. The extent to which teams could implement this strategy was predicted by the fluctuations in their scanning and movement behaviours, which correlated with changes in situation awareness due to experimentally varying the information that is deprived [21]. Further, work has been done to quantify how teams structure their verbal communication [22] as a function of information deprivation, which informs the design of synthetic teammates that can communicate verbally [44] and act adaptively [45] alongside human teammates.

## 6. Future considerations in human-autonomy teaming

Advancements in technologies that facilitate the training of artificial agents using machine learning, including reinforcement learning and generative AI, present new opportunities, but also challenges, in designing agents that will interact with humans in human-autonomy teaming [46]. One key concern is ensuring that the policies embodied by artificial agents, posing as synthetic teammates, are compatible with human co-actors. Here, compatibility refers to the extent to which the behaviours and decisions of artificial agents comply with either the expectations of human co-actors, or that minimally they can be predictable to facilitate coordination.

A major challenge in creating these artificial agents is that these methodologies are dependent on access to training datasets that may not be readily available. Again, one possible solution is to use synthetic simulation to generate sufficient training datasets to facilitate agent learning, a common approach in areas such as robotics [47] and autonomous driving [48]. However, due to the sample inefficiencies inherent in training agents using procedures that require high-speed simulation like deep reinforcement learning, humans may have to cope with a future where the agents they interact with may include experiences that are virtually created and evaluated [49] outside the scope of human capability. Accordingly, these simulated worlds may not accurately reflect the behaviours and predispositions of humans, resulting in potential conflicts during deployment. Alternatively, data-driven approaches may produce seemingly human-compatible behaviours that result in overtrust and misuse [50], and where coordination deteriorates when agents experience states that are outside the training distribution [37]. In team-based tasks, coordination deterioration is associated with a loss of situation awareness [21,51].

To combat these challenges, one approach to establishing trust [50,52] in these systems is to constrain their behaviours using well-defined, low-dimensional dynamical rules that emulate the dynamics of human behaviour and are reproducible. Research investigating both the dynamics of human small group interactions and non-human swarm systems have demonstrated that individual agents following simple low-dimensional rules can result in complex behavioural phenomena when embedded within a group or collective. For human perceptual-motor behaviours, this review provides an illustration for how the behaviours of individual actors can be understood and modelled as low-dimensional dynamical systems. Moreover, this review demonstrates that these models can not only generate similar coordinative behaviours as observed in humans, but they also allow for the emergence of new behavioural strategies when the models are embedded in human-autonomous teams. Consistent with the central tenets of complexity science—complexity from simplicity—our research provides a demonstration for how the behavioural dynamics of human shepherding in small group contexts are consistent with the general principles of swarm systems. For heterogeneous swarms consisting of humans and artificial agents working together, however, it is important to appropriately consider and model the dynamics of human behaviour and decision-making.

## Data Availability

This article has no additional data.
